# SYNBIO^®^ Probiotic and Antioxidant Dietary Supplementation: Clinical Trial Evaluation of Potential Effects on Airline Flight Crew Members’ Well-Being

**DOI:** 10.3390/microorganisms11040924

**Published:** 2023-04-02

**Authors:** Maria Magdalena Coman, Maria Vittoria Micioni Di Bonaventura, Carlo Cifani, Stefania Silvi, Maria Cristina Verdenelli

**Affiliations:** 1 Synbiotec S.r.l., Spin-Off of UNICAM, Via Gentile III Da Varano, 62032 Camerino, Italy; 2 School of Pharmacy, Pharmacology Unit, University of Camerino, 62032 Camerino, Italy; 3 School of Biosciences and Veterinary Medicine, University of Camerino, 62032 Camerino, Italy

**Keywords:** probiotics, lactobacilli, elderberry, antioxidant, crew members, microbiota, well-being

## Abstract

The irregular lifestyle of airline crew members, wide/adverse job-related exposures, and the impact of temporary hypoxia on gut microbiota well-being have increased concern about the daily recommended dose of certain nutrients among flight crew. The aim of this study was to determine if daily consumption of a SYNBIO^®^ probiotics–elderberry extract supplement (ACTIVE) may contribute to the well-being of flight attendants. Forty healthy crew members enrolled in a double-blind, randomized, placebo-controlled study consumed one ACTIVE capsule/day or placebo for 30 days. Bowel well-being, health-related quality of life, and gastrointestinal tolerance were assessed by validated questionnaires. Saliva and fecal samples were analyzed to determine secretory immunoglobulin-A (sIgA) levels and to characterize gut microbiota composition, respectively. ACTIVE subjects presented a physiological improvement and a statistically significant higher Psychological General Well-Being Index (PGWBI) global score compared to PLACEBO subjects. The ACTIVE subjects showed significantly increased levels of lactobacilli and bifidobacteria compared to the PLACEBO group, while a significant increase in lactobacilli and a significant reduction in Enterobacteriaceae were registered when compared with the beginning of supplementation, confirming the persistence of probiotics in the gastrointestinal tract and the direct antagonism and competitive exclusion effects. Additionally, sIgA levels were significantly higher in the ACTIVE group compared to the baseline and to the PLACEBO group at the end of supplementation. The ACTIVE supplementation might be beneficial to airline crew members, improving their physiological state, their immune defenses, and the strength and efficiency of their gastrointestinal tract when responding to stressful conditions.

## 1. Introduction

The life of airline crew members has been subjected to many changes over the past decades. The increase in flight frequency, reduced layovers, a reduction in minimum rest time, increased flight time limitations, and the increased amount of ultra-long-range flight have brought serious concerns about the quality of life of this category of workers to the attention of the Flight Aviation Authorities. The strong impact that high exposure to cosmic radiation might have on chromosomic translocation and the incidence of melanoma and other types of cancer, as well as the impact of circadian rhythm disruptions on psychological and physiological well-being, has extended the attention of aeronautical medicine to pilots and flight attendants [1,2]. Airlines are required to provide an extensive medical examination to maintain flight licenses. 

Yearly, airline crew members are subjected to a cosmic radiation medical examination to verify the impact of ionizing radiation on the skin, blood, and eyes [3]. At the same time, constant disruption of the circadian rhythm, the highly irregular lifestyle, the lower quality of the food eaten on airlines, and the high impact of temporary hypoxia on gut microflora’s well-being suggest the importance of properly assessing the flight crew’s nutrition. 

In this context, modifications in gut microbiota induced by episodic changes in blood oxygen content may be relevant since some metabolic alterations modulated by the gut microbiota (obesity, metabolic syndrome, etc.) are commonly associated with obstructive sleep apnea [4]. An interesting study reported the existence of a radial composition distribution of microbiota associated with the distribution of oxygen and nutrients provided by the host tissue in both mice and humans [5]. In humans, the microbiota analyses showed an enhanced level of oxygen-tolerant organisms of the Proteobacteria and Actinobacteria phyla in the rectal mucosa compared with the feces, revealing the influence of oxygen concentration on the composition of gut microbiota [5]. Thus, it can be presumed that the discontinuous alterations in blood oxygen potentially related to obstructive sleep apnea induce fluctuations in oxygen levels in the gut microbiota, leading to a variation in its composition in terms of variety and abundance. The gastrointestinal micro-ecosystem is always changeable. This situation occurs because of the high sensitivity of microbial populations to host-induced physio-chemical and environmental factors such as antimicrobial agents, stress, redox potential, drugs, temperature, and nutrients [6,7]. The intestinal microbiota is also highly sensitive to oxygen tension [8] and correlated with atmospheric pressure [9].

Individuals who live for long periods at high altitudes may be susceptible to a hypobaric hypoxia I condition that causes various physiological variations in hematology [10,11] or gastrointestinal disorders [12]. Acute mountain sickness is a recurrent disturbance for military personnel and travelers at high altitudes. Among the various problems that arise, gastrointestinal issues are the most important, with diarrhea, acid formation, flatulence, vomiting, anorexia, and irritable bowel syndrome as some of the complications observed. Moreover, flatus expulsion is a very frequent infection at high altitude [13] due to hypobaric hypoxia stress and is mediated by local hormones (e.g., leptin and cholecystokinin) [14].

Nowadays, EASA (the European Aviation Safety Agency), through EU Regulation 859/2008 of 20 August 2008, has established specific requirements for aircraft operators under which they are obliged to assess crew exposure to cosmic radiation. If the level of exposure exceeds the value of 1 mSv/year, a series of actions should be considered, including exposure metering, the reduction of as much exposure as possible, and the dissemination of information related to the possible consequent risks of exposure and the limits for exposure to cosmic radiation for staff in case of pregnancy [15]. Aircraft operators must keep records of flight crew exposure values and communicate them to their members both annually and at the termination of employment. The data must be stored by the operator for up to 12 months after the termination of employment. Currently, research conducted by several scientific institutions and European carriers has not provided unanimous and conclusive evidence regarding the correlation between pathological events and the professional activity of civil aviation crews [16]. However, considering the biological damage caused by low doses of ionizing radiation can be only probabilistic (or stochastic), an increase in dose may enhance the statistical probability of observing pathological phenomena as years go by. Practically, for dose levels absorbed by civil aviation crews, the probability of biological damage is estimated to be extremely low [15]. In fact, epidemiological studies comparing populations exposed to ionizing radiation with those not exposed revealed no disease at doses up to 400 millisieverts (low dose rate or low-intensity instant dose) [17,18]. 

Dietary supplementation with probiotics and antioxidants could have a preventive effect on serious issues related to the quality of life of airline crew members and increase their physiological defenses, which is significant considering the strong impact that high exposure to cosmic radiation might have on the incidence of several types of cancer and the impact of circadian rhythm disruptions on psychological and physiological well-being. Nowadays, consumer interest in foods containing health-promoting ingredients such as fruit bioactive extracts is increasing. Polyphenols are the most abundant phytochemicals (vitamins, minerals, carbohydrates, and fiber) in several fruits, and they confer many beneficial effects to the products. They include mainly phenolic acids, flavonoids, coumarins, and tannins [19,20]. A diet rich in fruit provides nutrients that are vital for human health, with anthocyanins receiving attention due to their health-promoting properties and biological activities, such as antioxidant, anti-inflammatory, and antimicrobial activity.

Elderberry (*Sambucus nigra* L.) is used to improve vision and boost the immune system due to its antioxidant and hypocholesterolemic activity. Elderberry is a notable source of water-soluble anthocyanins, containing flavonols and phenolic acids that are noted free radical scavengers [21]. These anthocyanins could prevent damage to the body’s cells [22], including damage due to oxidative stress and lipid peroxidation [23]. Elderberries contain approximately 80% water and 15% carbohydrates, with the remaining proportion consisting of fiber, proteins, vitamins (vitamin A, discrete quantities of vitamin C, and some B vitamins), and ash. Minerals present include potassium, calcium, sodium, phosphorus, iron, copper, zinc, and magnesium [24]. Elderberry may prove beneficial as a healing cure for cold and flus, decreasing congestion and helping sweat out toxins [25]. In our previous study [26], the total content of anthocyanins and flavonoids in elderberries and their antioxidant activity were assessed. In addition, elderberry extract combined with a SYNBIO^®^ probiotic formulation [27,28] revealed higher antioxidant activity than the single extract, suggesting that probiotics potentiate the antioxidant activity of elderberry [26]. Based on this, the mentioned fruit extract was considered a good candidate for the design of new functional foods/beverages and nutraceuticals in combination with specific probiotic strains.

The present clinical study has been designed to determine whether daily consumption of a novel dietary supplement containing a combination of probiotic SYNBIO^®^ (Synbiotec Srl, Camerino, Italy) and elderberry extract confers beneficial effects on airline flight crew members. Bowel habits, physiological and psychological health, gut microbiota composition, and secretory immunoglobulin-A (sIgA) concentration (as an immune marker) were the main parameters monitored in these subjects. 

## 2. Materials and Methods

### 2.1. Design, Production, and Stability of the Dietary Supplement

The ACTIVE capsules containing SYNBIO^®^ and elderberry extract were produced following a validated production process using a capper/filler (model MS-6/N, MultiPharma, Florence, Italy). Each capsule contained 0.2 g of SYNBIO^®^ probiotic blend (*L. rhamnosus* 501^®^ + *L. paracasei* 502^®^; 1:1; Synbiotec Srl, Camerino, Italy) and 0.07 g of elderberry extract to reach a final bacterial concentration of 15 billion probiotics per daily dose (one capsule). 

Five different elderberry extracts were used to produce ACTIVE capsules: elderberry extract at 6.5% (A), 10% (B), and 13% (C) (provided by a company under a confidentiality agreement); elderberry flower and leaf extract at 0.3% (D) (ACEF, Fiorenzuola d’Arda, PC, Italy); and elderberry fruit extract (E) (ACEF). 

To further investigate the effect of various storage temperatures on the viability of the probiotic strains, the jars containing the probiotic capsules were stored at three different temperatures (+4 °C, +25 °C, and +40 °C) and samples were collected and analyzed every month.

### 2.2. Microbiological and Physico-Chemical Analysis

#### 2.2.1. Viability of Probiotic Strains in Dietary Supplements

Viable cell counts of the probiotic SYNBIO^®^ blend (mix of *L. rhamnosus* IMC 501^®^ and *L. paracasei* IMC 502^®^), expressed as CFU/capsule, were determined using the agar plate method. In an Easy MIX homogenizer (AES Laboratory, Bruz, France), each capsule sample was homogenized in physiological solution (0.9% NaCl). A series of ten-fold dilutions was prepared and 100 µL was spread on MRS agar (de Man, Rogosa, Sharpe-Oxoid, Basingstoke, Hampshire, UK). After 72 h of aerobic incubation at 37 °C, the bacterial counts were made. On a few colonies selected from those isolated from each capsule, Gram stains, morphological characterization, and molecular analyses (Randomly Amplified Polymorphic DNA (RAPD)) [28] were carried out.

#### 2.2.2. Water Activity and Moisture Content

The water activity of the probiotic dietary supplements was evaluated using an AQUALAB 4TE instrument from Decagon Devices (Hopkins Ct. Pullman, WA, USA). Moisture content was also determined using the Thermo-balance Ohaus (Nanikon, Switzerland).

#### 2.2.3. Antioxidant Activity

The antioxidant activity of each capsule formulation was investigated using the DPPH (2,2-diphenil-1-picrylhydrazyl) method described by Coman and co-workers [26]. Briefly, each sample was allowed to react for 90 min with fresh DPPH solution at room temperature in a dark place. The absorbance (A) of the resulting solution was measured at 517 nm, and a linear regression for the Trolox standards was outlined. Inhibition (%) was calculated using the formula I (%) = (A_blank_ − A_sample_)/A_blank_ · 100; a higher inhibition percentage reflects higher antioxidant activity.

### 2.3. In Vivo Study

#### 2.3.1. Subjects

**Figure 1:**
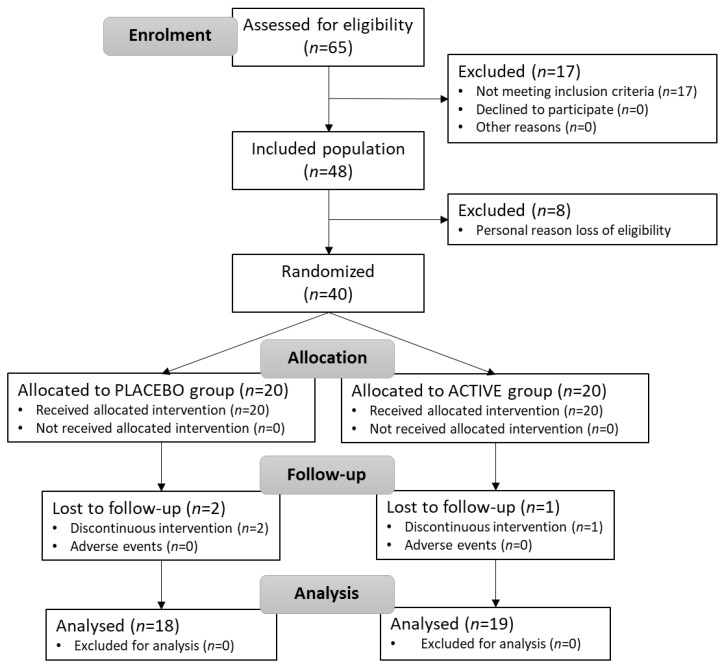
CONSORT subject flow diagram (PLACEBO group = control; ACTIVE group = probiotics–elderberry supplementation).

Forty consenting adults employed by a major airline company were enrolled in this randomized double-bind controlled human clinical trial and received daily placebo or probiotics–elderberry extract supplementation for 30 days (Figure 1). There were several inclusion criteria for the feasibility study: currently being employed as an airline flight crew member as either a pilot or flight attendant; being in good health; willingness to collaborate during the study; providing informed consent; and availability to complete questionnaires (surveys). Exclusion criteria included any history of gastrointestinal disorders; extreme types of constipation (less than two bowel movements a week); hypercolesterolemia (max. 250 mg/dL); any fruit allergy; treatment with antibiotics, sulfamidics, corticosteroids, or immunosuppressant agents; and use of laxatives, lactulose, probiotics or prebiotics in the past month/4 weeks. Moreover, there were also criteria for leaving the study, which occurred under the following conditions: non-continuity of study supplementation, severe illness, continuous use of antibiotics or laxatives, or consumption of other probiotics. 

The volunteers were divided into a PLACEBO group (6 females and 14 males) and an ACTIVE group supplemented with the probiotic and elderberry extract capsules (7 females and 13 males). 

#### 2.3.2. Dietary Supplement

Subjects allocated to the ACTIVE group received 1 capsule a day of lyophilized SYNBIO^®^ (*L. rhamnosus* 501^®^ and *L. paracasei* 502^®^; 1:1) (Synbiotec Srl) enriched with elderberry flower and leaf extract (ACEF, Fiorenzuola d’Arda, PC, Italy). Each capsule contained 0.2 g SYNBIO^®^ and 0.07 g/capsule elderberry flower and leaf extract, assuming a daily average intake of polyphenols of 50 mg/day; an extensive range of 30–100 mg/day was the daily recommended dose.

Subjects allocated to the PLACEBO group received 1 capsule a day containing 0.27 g of corn starch. The PLACEBO capsule was indistinguishable in color, smell, and taste from the probiotics capsule.

#### 2.3.3. Study Design

A single-center, double-blind, randomized, placebo-controlled study was designed and performed. Information exchange was established with competent medical institutions that confirmed that no interactions were possible between the content of the capsule and the toxicological urine tests that crew members have to randomly provide on a yearly basis due to requirements enforced by Italian Legislative Decree no. 81/08 Art.41, paragraph no. 4: “alla verifica della assenza di condizioni di alcoldipendenza e di assunzione di sostanze psicotrope e stupefacenti = to the verification of the absence of conditions of alcohol dependence and intake of psychotropic and narcotic substances”. The study protocol was conducted in accordance with the Declaration of Helsinki and approved by the medical institution of the airline company involved. All volunteers provided written informed consent before inclusion in the study.

#### 2.3.4. Sample Collection

Fecal and saliva samples were collected on day 0 before capsule consumption had started. Each subject was provided with 3 questionnaires: a bowel well-being questionnaire, the PGWBI (Psychological General Well-Being Index), and the GSRS (Gastrointestinal Symptom Rating Scale) [27]. The subjects were instructed to become familiar with the questionnaires so that they could fill them out at the end of the 30 days of treatment. A second set of fecal and saliva samples was collected at the end of the supplementation (day 30).

#### 2.3.5. Outcome Measures

##### Bowel Well-Being 

The subjects self-evaluated all the parameters at the end of the intervention. The subjects were asked to note whether, over the last 30 days, their intestinal well-being in terms of intestinal regularity and stool volume had remained unchanged, improved, or worsened compared to the period prior to starting supplementation. They were also asked to record the degree of change on a combined 10-point Likert scale (where −5 means strong worsening, 0 means no changes, and +5 means strong improvement). The ease of defecation, bloating, constipation, abdominal pain, intestinal cramps, feelings of incomplete defecation, incontinence, and halitosis were also examined as secondary outcome measures. Additionally, the change in each of these individual habits was evaluated with the same combined Likert scale [27]. Stool consistency was defined by the Bristol Stool Form Scale from type 1 to type 7 (type 1–2 indicates constipation, type 3–4 indicates ideal stools, and type 5–7 may indicate diarrhea or severe diarrhea) [29]. 

##### Health-Related Quality of Life

The Psychological General Well-Being Index (PGWBI) [30], a general questionnaire evaluating psychological well-being and distress, was used to evaluate the individuals’ health-related quality of life. It consists of 22 items that fall into six categories (anxiety, depression, self-control, positive well-being, general health, and vitality). A global score can be generated by combining multidimensional scores, with the global score ranging between 0 (worst) and 100 (best).

##### Gastrointestinal Tolerance

The Gastrointestinal Symptom Rating Scale (GSRS) [31] measured the gastrointestinal tolerance of the supplemented synbiotic product based on 15 questions, each of which was rated on a seven-point Likert scale from no discomfort (score 1) to very severe discomfort (score 7). A symptom score of two or higher on the GSRS was used to define intolerance after all 15 items of the questionnaire had been examined.

##### Modulation of Gut Microbiota Composition

Microbiota composition was assessed using a quantitative real-time PCR procedure (qPCR) for the quantification of selected bacterial groups from ACTIVE and PLACEBO subjects’ feces.

##### DNA Extraction from Fecal Samples

From the fecal samples of each subject collected before and at the end of the supplementation period, DNA was extracted using a Stool DNA Isolation kit (NorgenBiotek Corp., Thorold, ON, Canada) following a modified protocol according to the manufacturer’s instructions. Extracted DNA concentration and purity were checked using a NanoDrop ND-1000 Spectrophotometer (Thermo Scientific, Waltham, MA, USA) that operated at an excitation wavelength between 220 nm and 750 nm. The DNA samples were then stored at −20 °C until they were used for molecular analysis.

##### Quantitative Real-Time PCR 

A quantitative real-time PCR (qPCR) procedure was used for the quantification of selected bacterial groups from ACTIVE and PLACEBO fecal samples. The bacterial groups of interest were *Lactobacillus* spp., *Bifidobacterium* spp., *Staphylococcus* spp. *Bacteroides-Prevotella-Porphyromonas* spp., the *Clostridium coccoides–Eubacterium rectale* group, and Enterobacteriaceae. Using specific primers, parameters, and standard curves already created for each bacterial group, SYBR Green quantitative real-time PCR amplification was carried out using an iCycleriQ Real-Time Detection System (Stratagene, La Jolla, CA, USA) coupled with MxPro v. 3.00 software [32].

##### Recovery of Probiotic Strains from Fecal Samples

Fecal samples were examined for the presence of lactobacilli using MRS agar (de Man, Rogosa and Sharpe agar, Oxoid) and the application of a ten-fold serial dilution procedure to confirm colonization and the presence of both tested probiotic strains in the intestine [28]. Subsequently, 10–20% of the total colonies per sample, randomly chosen from countable agar plates after 48–72 h of aerobic incubation at 37 °C, were separated and tested for purity. DNA extracted from the selected colonies using a DNA extraction kit (NorgenBiotek Corp.) was analyzed using an RAPD technique [28].

##### Determination of sIgA in Saliva 

sIgA, which consists of two IgA monomers joined by the J-chain and an additional secretory component, is secreted in plasma cells located in the lamina propria of mucosal membranes; however, a lack of serum IgA does not necessarily correlate with a lack of sIgA. It plays a crucial role in the alternative complement pathway and in the activation of some inflammatory reactions. Determination of sIgA in the saliva of each subject was performed using the Human sIgA ELISA Kit (ImmunodiagnostikAG, Bensheim, Germany). The detection range was 0.156–10 ng mL^−1^, while sensitivity was <0.094 ng mL^−1^.

#### 2.3.6. Statistical Analysis 

The results are expressed as mean ± standard deviation or confidence limits. Comparison of the baseline characteristics of subject groups and of the mean absolute changes in the bowel habits frequency score, stool consistency, and PGWBI across the ACTIVE and PLACEBO groups was performed using Student’s *t* tests (significance level of *p* < 0.05). The same test was also applied to compare microbiological analysis results and those from the detection of secretory IgA in the saliva. Significant differences between mean values were determined by Tukey’s test after one-way analysis of variance using the GraphPad PRISM^®^ 8 program.

## 3. Results

### 3.1. Dietary Supplement Stability: Microbiological and Physico-Chemical Analysis

**Figure 2:**
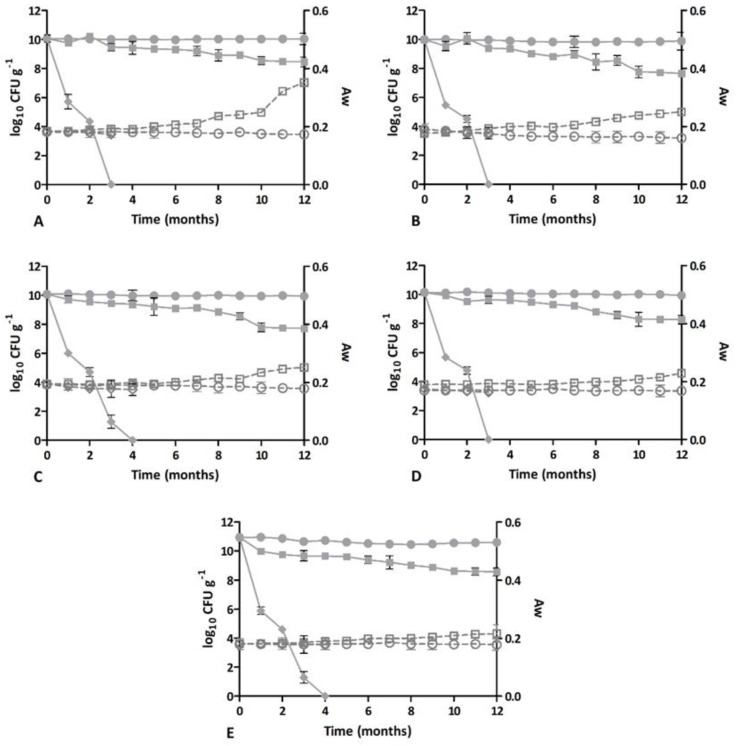
Trend over time of the bacterial count (expressed as log_10_ CFU capsule^−1^ (full sign)) and water activity (Aw—empty sign) of lyophilized SYNBIO^®^ powder combined with several elderberry extracts (elderberry extract at 6.5% (**A**), 10% (**B**), and 13% (**C**), elderberry flower and leaf extract at 0.3% (**D**), and elderberry fruit extract (**E**)) and stored at different temperatures (+4 °C (○), +25 °C (□), and +40 °C (◊)).

The trends for bacterial counts and the water activity of the five new formulations of capsules containing lyophilized SYNBIO^®^ probiotic powder and five different elderberry extracts stored at +4 °C, at room temperature (+25 °C), and at +40 °C are shown in Figure 2. It is remarkable that all formulations maintained their probiotic concentration for 12 months in refrigerated storage, while a decrease of less than 3 log was observed for capsules maintained at +25 °C. All the formulations had already exhibited a dramatic decrease after one month at +40 °C. Figure 2 also shows the water activity trends (Aw) of the five probiotic formulations, with slightly increased values for samples A, B, and C and no major changes in samples D and E. The same trend as Aw was noticed for moisture content and for antioxidant activity.

### 3.2. Participant Flow

Figure 1 describes the flow of subjects through the protocol. From the more than 100 contacted subjects, 65 showed interest in participating. Unfortunately, for different reasons, only 48 agreed to begin the clinical trial. The starting date was different for every subject according to their flying schedule. Before the start date, five subjects dropped out because of personal reasons and three needed to consume antibiotics. In the end, 40 subjects were included in the study and randomized (20 subjects into the PLACEBO group and 20 subjects into the ACTIVE group). Among them, 3 did not complete the 30 days of treatment (2 PLACEBO and 1 ACTIVE); therefore, 37 were included in the statistical analysis. The research product was unrelated to any subject withdrawals. Compliance with the study protocol was 100% across all subjects who successfully completed the trial in each study group. In both groups, subjects were evenly distributed with regard to the baseline features (Table 1).

### 3.3. Outcome Measures

#### 3.3.1. Bowel Well-Being

Primary outcomes such as intestinal regularity and stool volume are listed in Table 2. No significant differences were observed between the ACTIVE and PLACEBO groups over the four weeks of intervention regarding all assessed bowel habits (*p* > 0.05). Statistically significant differences (*p* < 0.05) were detected in the ACTIVE group after supplementation for intestinal regularity, stool volume, stool consistency, ease of defecation, rumbling of the stomach, swelling, flatulence, constipation, and diarrhea when compared to the starting point. Stool consistency was generally evaluated as type 4 and presented no significant difference between the two groups (Table 2). On the other hand, the subjects from the ACTIVE group also showed a significant improvement in terms of cold period and symptoms, fatigue, and weight variation at the end of probiotic supplementation compared to the baseline.

#### 3.3.2. Health-Related Quality of Life

Health-related quality of life was estimated according to Psychological General Well-Being Index (PGWBI) global scores, resulting in a mean value of 76.1 for the PLACEBO group and 84.1 for the ACTIVE group considering an evaluation range of 0 to 100 (best) (Table 3). Subjects in the supplemented group showed a statistically significant higher PGWBI global score, with a significant improvement in the management of anxiety and depression, general health status and vitality feeling, and general self-control with respect to the baseline.

As shown in Table 3, some parameters of the PGWBI are statistically higher in the ACTIVE group compared to the PLACEBO group at the end of 4 weeks of supplementation. In the PLACEBO group, a significant decrease in the management of depression and the general state of health was observed after the supplementation period.

This means that the general well-being of the subjects from the control group had a worsening trend compared to the subjects under ACTIVE supplementation, who expressed an improvement in most of the PGWBI parameters.

#### 3.3.3. Gastrointestinal Tolerance

The Gastrointestinal Symptom Rating Scale (GSRS) questionnaire, based on 15 items, revealed that the median score for each symptom in both the PLACEBO and ACTIVE groups was 1 (no discomfort) at the end of 30 days of supplementation (Table 4). There were no median scores of two or higher at the beginning of the PLACEBO/ACTIVE intervention. The GSRS scores showed the absence of intolerance to the supplementation treatment; thus, no serious adverse events during or after the supplementation period were observed in either the PLACEBO or ACTIVE group. However, the maximum score in the PLACEBO group was 7 (very severe discomfort), whereas the maximum score in the ACTIVE group was just 3 (moderate pain), indicating a potential preventive/beneficial effect from the ACTIVE supplementation.

As Table 4 shows, at the end of the 30-day supplementation period, no statistically significant differences were found between the PLACEBO and ACTIVE groups when compared to the baseline (*p* > 0.05, Student’s *t* test).

### 3.4. Gut Microbiota Modulation

#### 3.4.1. Bacterial Quantification by RT-PCR from Fecal Samples

Standard curves that were previously generated for the above-mentioned target strains of bacteria (*Bacteroides-Prevotella-Porphyromonas* spp., *Staphylococcus* spp., the *Clostridium coccoides–Eubacterium rectale* group, *Lactobacillus* spp., *Bifidobacterium* spp., and Enterobacteriaceae) were used for quantification through a quantitative qPCR procedure. 

The concentration of selected bacterial groups was detected for each fecal sample collected from the two experimental groups at the two different time-points of T0 and T30 (before and after supplementation with the ACTIVE or placebo treatment). 

**Figure 3:**
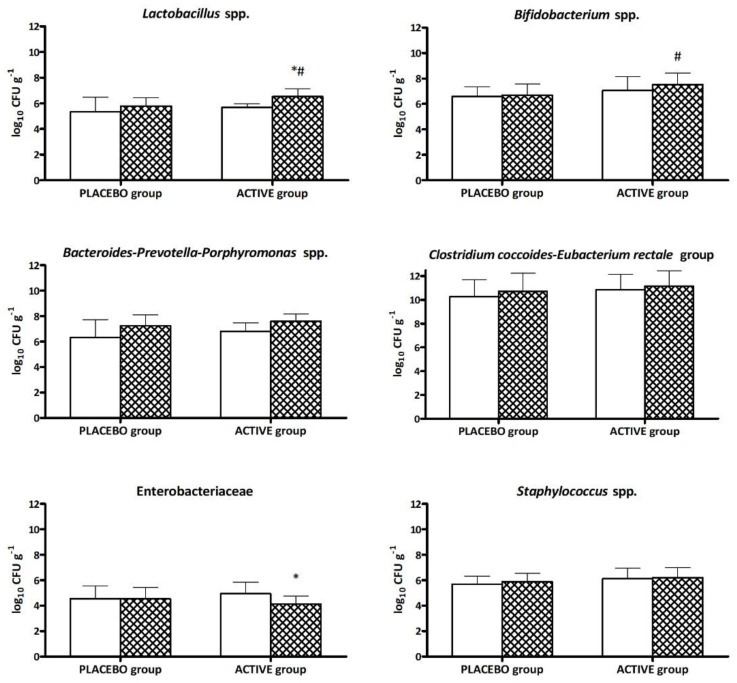
Fecal bacteria count (log_10_ CFU g^−1^ ± SD) of target bacterial groups during the different time points (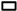 T0 and 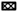 T30 days) relative to the two groups of volunteers (PLACEBO and ACTIVE). * Significantly different (*p* < 0.05) from T0 and ^#^ from the placebo group at the same time point according to Tukey’s test following one-way ANOVA.

Figure 3 shows the changes in microbiota composition over the supplementation period in both the ACTIVE and PLACEBO groups.

The probiotic supplementation significantly increased the *Lactobacillus* spp. level (Figure 3, *p* < 0.05) after 30 days of supplementation with respect to T0, while Enterobacteriaceae content decreased (*p* < 0.05), showing a potential direct antagonism and competitive exclusion effect provided by supplementation with probiotics [33]. Modulation of gut microbiota composition in subjects under ACTIVE supplementation compared to PLACEBO indicated a significant increase in *Lactobacillus* spp. and *Bifidobacterium* spp. levels (Figure 3, *p* < 0.05). No significant modification was found for the other bacterial groups (*Bacteroides-Prevotella-Porphyromonas* spp., *Staphylococcus* spp., and *Cl. coccoides-E. rectale* group) between the two groups during the supplementation period (Figure 3).

#### 3.4.2. Recovery of Probiotic Strains from Fecal Samples

This molecular analysis was carried out in order to confirm intestinal transit survival of probiotic strains in human subjects. The two probiotic strains of SYNBIO^®^ mix (*L. rhamnosus* 501^®^ and *L. paracasei* 502^®^; 1:1) were recovered in all the ACTIVE group fecal samples collected at the end of supplementation. The strains were not detected in any of the fecal samples collected before supplementation, nor in any of the PLACEBO group fecal samples collected after supplementation.

The average value of recovery percentages with respect to total *Lactobacillus* spp. was 24.87% for *L. rhamnosus* 501^®^ and 21.15% for *L. paracasei* 502^®^ at the end of the administration period.

### 3.5. Concentration of sIgA in Saliva

Table 5 shows the results of secretory IgA detection in the saliva of the two groups before and after 30 days of dietary supplementation. The results show a statistically significant difference (*p* < 0.05) between the two groups at T30 (11.2 ± 6.8 ng mL^−1^ for the PLACEBO group and 15.3 ± 6.7 ng mL^−1^ for the ACTIVE group). There was also a statistically significant increase (*p* < 0.05) in sIgA levels for the ACTIVE group after the 30 days of treatment (11.0 ± 7.2 ng mL^−1^ at T0 and 15.3 ± 6.7 ng mL^−1^ at T30).

## 4. Discussion

The professional activity of an airline flight crew member is often considered to be rather stressful. The increase in flight frequencies, reduced minimum rest time, disruption of the circadian rhythm, and the poor quality of food served onboard airplanes expose these subjects to a higher risk of dysbiosis, high cell oxidation, impaired immunity systems, and psychological stress. Being an airline flight crew member might seem fascinating, but the job comes with some side effects. During a working month, a flight crew member can lose up to 8–10 nights of sleep, and it is practically impossible to maintain regular eating habits during normal working hours. It is often complicated for these subjects to achieve a balanced diet in terms of macro- and micronutrients. The physiological impact of the job is raising concerns about the daily recommended intake of certain nutrients among flight crews. Compared to the general population, flight attendants have an increased prevalence of intestinal disorders and oxidative stress associated with low immune system function.

The concepts of healthy gut microbiota, gastrointestinal tract and oxidative stress, and their involvement in different disease conditions represent our goal in the present study, which was to evaluate the potential beneficial effects of a new dietary supplement formulation (SYNBIO^®^ + elderberry extract) in these workers. 

Bowel well-being was one of the primary outcomes, though consideration was given to the fact that bowel habits and well-being can be perceived in different ways since, in many cases, it is essentially a matter of self-perception. In our surveys, we assessed parameters such as intestinal regularity, stool volume, ease of defecation, the frequency of bloating, constipation, and the sensation of complete defecation. These are regarded as indicators of ideal bowel function because they ostensibly prevent the prolonged residence of stools in the colon, thus improving small bowel digestion and large bowel fluid and electrolyte re-absorption. In our study, self-evaluation did not lead to significantly different scores between the two groups, which we partially expected due to the short duration of the supplementation. On the other hand, we had a surprising positive result regarding constipation and the related intestinal parameters (intestinal regularity, stool volume and consistency, flatulence, etc.), from which some subjects of the ACTIVE group were suffering at T0 though not at T30.

A general sensation of “feeling good” was also evaluated through a questionnaire using the PGWBI, which is a widely used scale across many medical specialties in many countries [30]. The results indicated a significantly higher PGWBI score for participants in the ACTIVE group taking the probiotic–elderberry dietary supplementation.

The safety of the supplementation was another important matter that was considered. The supplementation proved to be safe, as no adverse events were reported when the subjects provided information in the GSRS questionnaire [31]. A few cases of severe discomfort (scores of 7) were highlighted in the PLACEBO group and only a few cases of mild discomfort (scores of 3) were observed in the ACTIVE group, thus suggesting a potential preventive/beneficial effect of supplementation.

The distal gut microbiota of healthy humans is considerably diverse and possibly contains over 1000 species. The phyla Firmicutes and Bacteroidetes appear to determine the existence of a shared core microbiome in healthy individuals [34]. Within each unique microbial community, the relative quantities and species present might change drastically. According to other research defining the microbial gene profile, there are functional pathways that are common to all persons (a functional core microbiome), although the pathway may be filled by various types or communities of bacteria depending on the individual [35]. Low diversity was seen in patients with recurrent *Clostridium difficile* infections, while high diversity in the gut microbiota appeared to be related with good health [36,37]. Moreover, the metabolic profiles of various microbial communities or the relative quantities of particular types of bacteria may have an impact on human health [38]. Based on these above-mentioned criteria, *Lactobacillus* spp., *Bifidobacterium* spp., Enterobacteriaceae, *Bacteroides-Prevotella-Porphyromonas* spp., the *Cl. Coccoides–Eu. rectale* group, and *Staphylococcus* spp. were the main bacterial groups of interest in our study. The achieved results showed a homogeneous microbiota composition at T0 in all participating subjects. After the 30 days of daily supplementation, the subjects in the ACTIVE group showed a significant CFUg^−1^ increase in *Lactobacillus* and *Bifidobacterium* species in comparison with the PLACEBO group, confirming the ability of SYNBIO^®^ to colonize and persist in the gastrointestinal tract [27,39,40,41,42]; the appreciable reduction in Enterobacteriaceae highlights and confirms the probiotic capacity of exerting a direct antagonism and a competitive exclusion of pathogens [33,43]. The positive modulation of intestinal microbiota strongly impacted global PGWBI scores for the probiotic–elderberry supplemented group (ACTIVE).

Our last primary outcome concerned oxidative stress and immunological response due to the high impact of ionizing radiation and the increasing number of specific diseases among flight crew members. Saliva sIgA, assessed through an ELISA kit, is the main immunoglobulin found in mucus secretions, and it neutralizes mucosal pathogens [44,45] whilst exhibiting various recognized functions [46]. It is identified as a reliable marker of mucosal protection against enteric disease [47] and of immunological stress [48]. Further, it is typically considered the first line of defense from environmental factors [49]; this immunoglobulin is also a reliable biomarker for the evaluation of physiological stress [50,51]. In our study, we detected a significant increase in sIgA in the saliva of ACTIVE group volunteers after the 30 days of supplementation. This increase was considered as a marker of enhancements to the body’s capacity to defend itself, which was likely due to the beneficial action induced by the high content of antioxidant polyphenols present in the elderberry extract and their potential combination with probiotics.

In conclusion, this is the first study to our knowledge that assesses the potential beneficial effects of a combination of probiotics and elderberry extract on airline flight crew members. The results demonstrated the significant difference in specific groups of bacteria counts for all the subjects who self-administered the ACTIVE formulation daily. The beneficial effect was established by evidence indicating improved persistence and colonization of *Lactobacillus* and *Bifidobacterium* strains, the absence of disease and side effects during the trial period, and the appreciable increase in sIgA, confirming a strong physiological response in the immune system. 

The combination of both SYNBIO^®^ and elderberry extract proves to be safe and efficient and allows this work to act as a pilot study for further scientific evaluation involving a larger number of subjects for a longer period of supplementation exposure.

The SYNBIO^®^ and elderberry extract dietary supplementation might be beneficial for airline crew members as it can increase their overall physiological state, improve their defenses, and increase the strength and efficiency of their gastrointestinal apparatus in response to the continues exogenous attacks they are subjected to during their highly demanding working life.

## Figures and Tables

**Table 1:** Baseline characteristics of subjects: comparison between the two experimental groups.

Characteristics	PLACEBO (*n* = 18)	ACTIVE (*n* = 19)	*p* *
Age, years (mean ± confidence limits)	44.3 ± 1.8	46.6 ± 3.0	>0.05
Male	45.6 ± 1.6	47.9 ± 4.1	>0.05
Female	42.4 ± 3.5	43.7 ± 3.1	>0.05
Gender			
Male (*n* [%])	11 (61)	13 (68)	>0.05
Female (*n* [%])	7 (39)	6 (32)	>0.05
Current smokers			
No (*n* [%])	15 (83)	12 (63)	>0.05
Yes (*n* [%])	3 (17)	7 (37)	>0.05
Overweight/obese			
No (*n* [%])	17 (94)	16 (84)	>0.05
Yes (*n* [%])	1 (6)	3 (16)	>0.05

PLACEBO: control group; ACTIVE: probiotic–elderberry supplementation group. * The significance level is *p* < 0.05 according to the Student’s *t* test.

**Table 2:** Mean absolute changes in bowel habits and other health parameters and stool consistency type over the 4 weeks of the supplementation period.

Bowel Habits and Other Health Parameters *	PLACEBO (*n* = 18) (Mean ± Confidence Limits)	ACTIVE (*n* = 19) (Mean ± Confidence Limits)
Intestinal regularity	0.4 ± 0.3	1.2 ± 0.2 ^†^
Stool volume	0.1 ± 0.2	1.2 ± 0.3 ^†^
Stool consistency	0.4 ± 0.3	0.9 ± 0.2 ^†^
Ease of defecation	0.6 ± 0.3	1.3 ± 0.2 ^†^
Rumbling of the stomach	0.1 ± 0.2	0.7 ± 0.2 ^†^
Swelling	0.3 ± 0.2	0.7 ± 0.1 ^†^
Flatulence	0.5 ± 0.3	0.8 ± 0.2 ^†^
Constipation	−0.1 ± 0.3	0.8 ± 0.2 ^†^
Diarrhea	0.1 ± 0.1	0.5 ± 0.1 ^†^
Abdominal pains	0.2 ± 0.1	0.3 ± 0.1
Intestinal cramps	0.2 ± 0.1	0.2 ± 0.1
Headaches	−0.1 ± 0.0	0.1 ± 0.1
Toothache	0.1 ± 0.3	0.3 ± 0.0
Dermatitis	0.0 ± 0.0	0.3 ± 0.0
Skin rash	0.1 ± 0.0	0.2 ± 0.0
Itch	−0.1 ± 0.1	0.4 ± 0.1
Cold	0.1 ± 0.2	0.6 ± 0.1 ^†^
Fever	0.1 ± 0.0	0.5 ± 0.1
Fatigue	0.2 ± 0.2	0.7 ± 0.1 ^†^
Weight variations	0.1 ± 0.0	0.5 ± 0.1 ^†^
Vomit	0.0 ± 0.0	0.2 ± 0.0
Nausea	0.0 ± 0.0	0.1 ± 0.0
Incontinence	0.0 ± 0.0	0.2 ± 0.0
Halitosis	0.1 ± 0.0	0.3 ± 0.0 ^†^
Food intolerance	0.2 ± 0.0	0.3 ± 0.0
**Stool consistency ^#^**		
Type	4 ± 0.5	4 ± 0.4

* Changes in bowel habits were assessed with a ten-point combined Likert scale (−5, 0, +5). ^#^ The assessment was conducted using the Bristol Stool Chart. ^†^ Significantly different from the starting point of supplementation (T0; Student’s *t* test, *p* < 0.05).

**Table 3:** Health-related quality of life assessed through global scores for the Psychological General Well-Being Index (PGWBI) questionnaire in both groups (PLACEBO and ACTIVE) at baseline and at the end of the double-blind supplementation period.

	Global Score * (Mean Values ± Confidence Limits)					
	PLACEBO			ACTIVE		
	Baseline	Week 4	Change Δ	Baseline	Week 4	Change Δ
**Psychological–emotional** **health** (negative aspects)						
Management of anxiety	73.8 ± 3.4	73.3 ± 3.3	−0.4 ± 0.3	75.8 ± 2.2	81.0 ± 2.4	5.2 ± 1.5 ^†^
Management of depression	85.3 ± 2.5	82.5 ± 2.7	−2.8 ± 0.8 ^†^	90.8 ± 1.6	94.9 ± 1.2	4.1 ± 1.2 ^†‡^
**General health** (intermediate aspects)						
General health	87.1 ± 1.0	85.9 ± 1.1	−1.2 ± 0.4	82.1 ± 1.4	88.1 ± 1.9	6.0 ± 1.8 ^†^
Vitality	66.7 ± 2.1	66.9 ± 2.0	0.3 ± 0.5	71.8 ± 2.1	77.6 ± 2.1	5.8 ± 1.4 ^†‡^
**Psychological–emotional** **health** (positive aspects)						
Positivity	66.4 ± 2.5	65.8 ± 2.5	−0.6 ± 0.2	69.3 ± 2.4	72.4 ± 2.1	3.1 ± 1.5
Self-control	83.3 ± 3.0	82.2 ± 3.0	−1.2 ± 0.4	82.5 ± 2.9	90.7 ± 1.4	8.2 ± 2.3 ^†^
**General state of health** (total score)	77.1 ± 2.0	76.1 ± 2.0	−1.0 ± 0.2 ^†^	78.7 ± 1.7	84.1 ± 1.6	5.4 ± 0.5 ^†‡^

* The global score ranged from 0 to 100 (best); Δ—difference between week 4 and baseline. ^†^ Significantly different from the starting point of supplementation (T0; Student’s *t* test, *p* < 0.05). ^‡^ Significantly different from the PLACEBO group (Student’s *t* test, *p* < 0.05).

**Table 4:** Gastrointestinal symptom scores according to Gastrointestinal Symptom Rating Scale (GSRS) in the two experimental groups expressed as median value, range, and mean values ± standard deviation at the end of supplementation.

Symptom *	PLACEBO (*n* = 18)			ACTIVE (*n* = 19)		
	Median	Range	Mean ± SD	Median	Range	Mean ± SD
Stomach ache or pain	1	1–3	1.17 ± 0.51	1	1–2	1.05 ± 0.23
Heartburn	1	1–2	1.11 ± 0.32	1	1	1.00 ± 0.00
Acid reflux	1	1–2	1.11 ± 0.32	1	1-2	1.11 ± 0.32
Hunger pains in the stomach/belly	1	1–4	1.17 ± 0.71	1	1	1.00 ± 0.00
Nausea	1	1–2	1.06 ± 0.24	1	1	1.00 ± 0.00
Rumbling stomach/belly	1	1–4	1.39 ± 0.85	1	1	1.00 ± 0.00
Bloated stomach/belly	1	1–5	1.61 ± 1.33	1	1–3	1.16 ± 0.50
Burping	1	1–3	1.22 ± 0.55	1	1–2	1.11 ± 0.32
Passing gas or flatus	1	1–7	2.11 ± 1.71	1	1–3	1.42 ± 0.69
Constipation	1	1–2	1.11 ± 0.32	1	1–2	1.11 ± 0.32
Diarrhea	1	1–3	1.39 ± 1.04	1	1–2	1.05 ± 0.23
Loose stools	1	1–6	1.44 ± 1.25	1	1–2	1.05 ± 0.23
Hard stools	1	1–3	1.11 ± 0.47	1	1–2	1.05 ± 0.23
Urgent need (bowel movement)	1	1–6	1.50 ± 1.20	1	1–2	1.11 ± 0.32
Feeling of not completely emptying	1	1–7	1.83 ± 1.86	1	1–3	1.21 ± 0.54

* Gastrointestinal symptoms were assessed with a seven-point Likert scale from no discomfort (1) to very severe discomfort (7) using the GSRS questionnaire.

**Table 5:** sIgA concentration in the saliva (ng mL^−1^) of subjects from the two groups studied before and after dietary supplementation.

	PLACEBO		ACTIVE	
	T0	T30	T0	T30
**sIgA** (ng mL^−1^) *	11.30 ± 6.39	11.18 ± 6.77	10.95 ± 7.19	15.25 ± 6.73 ^†‡^

* Mean values ± standard deviation; PLACEBO: control group; ACTIVE: probiotic–elderberry group; T0: before treatment; T30: after 30 days of treatment; ^†^ statistically significant difference from the respective T0 value (Student’s *t* test); ^‡^ statistically significant difference from the control group at T30 (Student’s *t* test); *p* < 0.05.

## Data Availability

Raw data were generated at Synbiotec S.r.l., derived data supporting the findings of this study are available from the corresponding and senior authors MMC and MCV on request.
